# Retraction: A. Amedei et al. Multiple Sclerosis: The Role of Cytokines in Pathogenesis and in Therapies. *Int. J. Mol. Sci.* 2012, *13*, 13438–13460

**DOI:** 10.3390/ijms17071021

**Published:** 2016-07-04

**Authors:** International Journal of Molecular Sciences Editorial Office

**Affiliations:** MDPI AG, Klybeckstrasse 64, 4057 Basel, Switzerland; E-Mail: ijms@mdpi.com

We have been made aware that the title paper [1] contains text taken verbatim from previously published articles by Shyi-Jou Chen et al. [2] and Ghislain Opdenakker and Jo Van Damme [3]. MDPI is a member of the Committee on Publication Ethics and takes the responsibility to enforce strict ethical policies and standards very seriously. To ensure the addition of only high quality scientific works to the field of scholarly publication, paper [1] is retracted and shall be marked accordingly. We apologize to our readership that this went undetected until now.
